# Risk factors associated with Hepatitis C among female substance users enrolled in community-based HIV prevention studies

**DOI:** 10.1186/1756-0500-4-126

**Published:** 2011-04-14

**Authors:** Diana Nurutdinova, Arbi B Abdallah, Susan Bradford, Catina C O'Leary, Linda B Cottler

**Affiliations:** 1 St. Louis VA Medical Center, 915 North Grand Blvd, St. Louis, MO, 63106 USA; 2 Department of Psychiatry, Washington University School of Medicine, 40 N. Kingshighway, Suite 4, St. Louis, MO 63108 USA; 3 Department of Medicine, Washington University School of Medicine, St. Louis, MO, USA

## Abstract

**Background:**

Hepatitis C virus (HCV) infection is one of the most frequent chronic blood-borne infections in the United States. The epidemiology of HCV transmission is not completely understood, particularly in women and minorities.

**Findings:**

We examined the HCV associated risk factors in substance abusing females involved in National Institute on Alcohol Abuse and Alcoholism (NIAAA) and National Institute on Drug Abuse (NIDA) funded HIV prevention studies of street recruited women. As a part of the 12 month follow-up, participants were interviewed about substance use and sexual risk behaviors, including drug implement sharing practices, tattoos, body piercing and blood transfusions and the sharing of personal hygiene equipment including tweezers, toothbrushes and razors. Urine and blood testing for HCV antibody (Ab), HIV and sexually transmitted diseases (STDs) was conducted at the time of assessment.

Among 782 predominantly African American women, 162 (21%) tested positive for HCV Ab. Older age (p < 0.001), history of injection drug use (p < 0.001), lifetime crack cocaine use (p = 0.004) and having a tattoo (p = 0.01) were significantly associated with HCV Ab positivity. Other risk factors previously reported in association with HCV Ab positivity such as sexual risk behaviors were not significantly associated with the presence of a positive HCV Ab.

**Conclusions:**

This large community based sample of predominantly African American substance abusing women showed high prevalence of HCV Ab positivity and low awareness of their HCV serostatus. Our study demonstrated that in addition to intravenous drug use (IDU), other factors were significantly associated with HCV Ab positivity such as having a tattoo and a lifetime history of crack use. Other potential routes of HCV transmission should be further studied among high risk female populations.

## Background

Hepatitis C virus (HCV) infection is one of the most frequent chronic blood-borne infections in the United States, causing significant morbidity and mortality. An estimated 4500 in-hospital deaths per year are related to the HCV infection and associated liver disease in the United States [1]. Based on the most recent data from the National Health and Nutrition Examination Survey III (NHANES III) cohort, the general population prevalence of HCV antibody (Ab) is 1.6% with 1.3% or 3.2 million persons chronically infected with HCV [2]. Due to its chronic nature, HCV infection often leads to an end-stage liver disease that may subsequently require liver transplantation. HCV infection is the most common indication for liver transplantation in the US; between 15 and 50% of liver transplantations are performed due to HCV infection [3].

HCV was originally documented as a non-A non-B virus causing transfusion related hepatitis [4]. HCV is frequently spread through parenteral exposure and has been commonly associated with intravenous drug use (IDU). Introduction of universal blood screening in the US in 1990 led to a significant decrease in the risk of transfusion associated HCV infection [5].

The prevalence of HCV infection varies depending on age, race and certain risk factors. Previous epidemiologic studies have shown that there is a higher prevalence of HCV in African Americans, for reasons that are not clearly identified [6-9]. Prevalence of HCV among females is lower compared to males regardless of race [10], though HCV among females has been most extensively studied in the setting of HIV coinfection focused on the risk of vertical transmission. Among the risk factors associated with the HCV infection, drug injection remains a predominant route of transmission. Among IDU's, HCV prevalence ranges from 60-90% and carries a high morbidity burden [11].

Sexual transmission of the HCV is possible but much less efficient as compared to hepatitis B virus or HIV [12]. The risk of sexual transmission of HCV appears to be increased in the presence of HIV [13,14]. Vertical transmission occurs in an estimated 6-7% in the presence of viremia [15]. Routes of transmission other than IDU have been proposed and include tattoos, body piercings, shared drug preparation equipment and shared personal hygiene items [16-18]. The prevalence of HCV infection is higher in non-injection drug users than in the general public [19,20]; intranasal cocaine use and sharing of drug-use equipment has been associated with risk of HCV infection [21]. Additional virologic data confirming presence of HCV in nasal secretions supports this association [22].

A limited number of epidemiological studies have comprehensively examined the risk factors associated with HCV in women, particularly in underrepresented female populations. We examined HCV associated risk factors in a sample of substance abusing predominantly African American females involved in two HIV prevention studies conducted by the Epidemiology and Prevention Research Group (EPRG) at Washington University School of Medicine.

## Methods

### Sample and Study Description

The data are derived from the two NIH funded HIV prevention studies, focused on providing community based interventions in St Louis, Missouri: Women Teaching Women (WTW, n = 501), funded by NIDA, and the NIAAA funded Sister To Sister (STS, n = 348) conducted between 1998 and 2004.

Women for both studies were recruited by Community Health Outreach Workers (CHOWS) via street outreach [23,24]. The targeted sampling plan identified areas for recruitment based on the rates of HIV, drug use and sex trading reported from both community surveillance and law enforcement task force reports. Both projects were conducted at a satellite office located in the outreach area, established in 1989 and sustained with NIH funding [24,25].

Women were considered eligible if they were 18 years or older, sexually active in the 12 months prior to entering the study, and did not undergo either alcohol or substance abuse treatment 30 days prior to the study enrollment. WTW enrolled women who tested positive for cocaine, amphetamines or opiates by urine drug screening and/or had fresh "track marks", indicative of current intravenous drug use. STS targeted female heavy or problem alcohol drinkers who did not test positive for any substance by urine drug screening. Problem or heavy alcohol use was determined by a score of four or greater on the Alcohol Use Disorders Identification Test (AUDIT), a 10-item questionnaire assessing heavy alcohol consumption [26].

Structured interviews were conducted by trained non-clinician interviewers. The baseline assessments included a Washington University Risk Behavior Assessment for Women (WU-RBA-W), Substance Abuse Module (SAM), and a Diagnostic Interview Schedule (DIS) Version IV [27]. The WU-RBA-W was modeled after the NIDA Cooperative Agreement RBA that has an established test-retest reliability and validity [28,29]; it was specifically tailored to assess risky sexual and other behaviors among women. The SAM is a structured interview developed as an expanded version of WHO CIDI [30] that covers substance use, use and dependence on 11 drug categories, alcohol and tobacco [31]. The DIS assessed DSM IV lifetime and current psychiatric disorders (depression, conduct disorder, antisocial personality disorder, post traumatic stress disorder and pathological gambling).

In addition to interviews at baseline each woman met with the Peer Facilitator to receive the NIDA HIV pre and post-test counseling [32], a blood draw for hepatitis C antibody (HCV Ab), HIV antibody, Rapid plasma reagin (RPR) syphilis test, and urine testing for Gonorrhea and Chlamydia. Participants were then randomized into the intervention groups. In addition to the standard HIV intervention (HIV pre and post test counseling), approximately half of the women were assigned to a Well Woman examination, with and without four peer delivered sessions. Further details of the intervention in both STS and WTW have been presented elsewhere [33].

Participants were followed up at 4- and 12- months post intervention with repeat assessments including the WU-RBA-W with a trailer containing additional questions on sharing drug use equipment, tattoos, body piercings and blood transfusions, the SAM and DIS. Repeat laboratory testing for HCV Ab, HIV, RPR, Chlamydia and Gonorrhea was performed at the 12-month follow-up.

The data reported in this paper are from the combined STS/WTW sample collected at the 12-month follow-up, as it contained more detailed information on risk factors traditionally associated with blood-borne infections such as HCV than the baseline and 4-month interviews. Participants were consented to join the studies; all procedures were approved by the Washington University Human Research Protection Office (HRPO, study numbers 97-0438 and 98-0374), and were compliant with federal Health Insurance Portability and Accountability Act (HIPAA) regulations; confidentiality was further assured NIH Certificates of Confidentiality (NIDA and NIAAA).

### Laboratory Testing

Blood samples obtained at the baseline and 12-month follow-up were tested for HIV, HCV and syphilis. Antibodies to HCV (HCV Ab) were detected with a third-generation enzyme immunoassay (EIA-2.0; Abbott Laboratories, Abbott Park, IL). Further confirmatory assay was not performed because HCV Ab testing by EIA had a high positive predictive value in this high risk population. HIV testing was conducted with enzyme immunoassay and confirmed by Western Blot. Blood samples were also tested for syphilis and urine specimens were collected for chlamydia and gonorrhea testing.

### Statistical Analyses

Samples of the STS and WTW studies were combined for the statistical analyses because women were recruited in the same high risk areas and during overlapping periods. HIV positive participants were not randomized in either study and were therefore excluded from the sample (n = 19). Participants who did not complete all baseline assessments or did not have HCV Ab results were also excluded from this sample (n = 88), resulting in a total sample of 816 women (STS: n = 348; WTW: n = 501) for baseline and 782 women for the follow-up. In addition, there were 4 incident HCV Ab positive test results at follow-up testing that were excluded from the analysis.

The women were analyzed based on their HCV Ab status using composite variables with regards to the following categories: demographics, lifetime substance use and reported risky behaviors. Variables were then tested for association with HCV Ab positivity using the SAS ordered logistic regression procedure; odds ratios (OR) and 95% confidence intervals (CI) were calculated in four consecutive models. Variables found to be significantly related to HCV Ab positivity in initial model were included in the next model; each subsequent model contained variables significant from the previous model. This approach was chosen to avoid co-linearity of the variables and complex interactions in the models. SAS^® ^statistical package release 9.1 (SAS Institute, Inc. 1999) was used for bivariate (chi-square) analyses of the association between the dependent variable of HCV Ab status and each of the socio-demographic and independent categorical variables. Mean values were compared using t-tests. All tests were two tailed with a significance level defined as p < 0.05.

## Results

Using street outreach techniques, more than 5500 women in the target recruitment area were approached by CHOWS about participation in the studies. Further eligibility screening yielded 935 women; of those, 816 women were interviewed at baseline and 782 presented for the 12-month follow-up assessments and had HCV Ab test results available (96% follow-up rate).

At baseline, 173 out of 816 women tested positive for HCV Ab for a prevalence of 21.2% with similar rate at the 12-month follow-up (n = 162, 20.7%). However, at baseline only 70 women (40%) recalled that they were previously told by a health care provider that they had had "viral hepatitis", meaning that the majority of women were not previously tested and learned about their positive HCV Ab test for the first time through the study.

### Baseline characteristics

Baseline sociodemographic characteristics of women recruited in STS/WTW are shown in Table 1, which shows comparisons according to women's HCV Ab status. The overall sample was 83% African American while 77% of the HCV Ab positive women were African American. Women in HCV Ab positive sample were significantly different from HCV Ab negative women; HCV Ab positive women compared to HCV Ab negative women tended to be older, currently married, less educated and reported history of prior arrests.

**Table 1:** Sociodemographic characteristics at baseline and follow-up assessments

**Characteristics**	**Baseline** **N = 816 (%)**	**12 Month** **N = 782 (%)**	**12 Month Follow-up** **N = 782 (%)**		
			**HCV Ab (-)** **N = 620**	**HCV Ab (+)** **N = 162**	**P value**
*Age*					
Mean Age, years (±SD)	35.8 ± 9.0	37.2 ± 9.0	35.9 ± 8.9	42.3 ± 7.0	<0.004
37 years or older	455 (56%)	417 (53%)	294 (47%)	123 (76%)	<0.001
*Race*					
Non African American	147 (18%)	132 (17%)	529 (85%)	121 (77%)	0.001
*Marital status*					
Never married	532 (65%)	469 (60%)	394 (63%)	75 (46%)	<0.001
*Children*					
More than one child	649 (79%)	647 (83%)	507 (82%)	140 (86%)	NS
*Education level*					
Less than high school	443 (54%)	415 (53%)	315 (51%)	62 (62%)	0.01
education					
*History of arrest (ever arrested)*	604 (74%)	593 (76%)	448 (72%)	145 (89%)	<0.001
*Employment status*					
Did not work in past 12 months	249 (30%)	354 (45%)	291 (47%)	63 (39%)	NS
*Considered themselves homeless*	486 (60%)	576 (74%)	456 (73%)	120 (74%)	NS

### Substance Use and Sexual Behaviour

The risk for HCV Ab positivity was associated with substance use and sexual risk behaviors as shown in Table 2. HCV Ab positive women were also more likely to meet DSM-IV criteria for opiate and alcohol dependence at baseline assessment (26% vs. 51%, p = 0.001 and 52% vs. 61%, p = 0.044 respectively). HCV Ab positive women reported using more drugs than their HCV negative counterparts; they were more likely to undergo drug treatment, report binge drinking and attend AA meetings. The majority of HCV Ab positive women (69%) reported a history of IDU. HCV Ab positive injectors were more likely to report reusing needles and syringes than HCV Ab negative injectors (p = 0.0293). There was no significant difference in reusing a cooker, cotton or rinse water or in obtaining needles from friends and or a pharmacy. HCV Ab positive women were more likely to smoke crack cocaine than HCV Ab negative women (94% vs. 71%, p < 0.001). Among cocaine users, HCV Ab positive women were more likely to reuse crack cocaine related equipment compared to HCV Ab negative women.

**Table 2:** Substance use and risky behaviours reported at follow-up assessment

**Characteristics**	**HCV negative** ** N = 620 (%)**	**HCV positive** ** N = 162 (%)**	**p value**
**Substance Use**			
Reused needles/syringes*	14 (38%)	61 (59%)	0.03
Reused cooker/cotton/rinse water*	11 (30%)	49 (47%)	NS
Gotten needles from friends/pharmacy*	30 (81%)	94 (89%)	NS
Lifetime crack cocaine use	442 (71%)	152 (94%)	<0.001
Reuse straight shooter/crack pipe belonging to others^a^	288 (65%)	119 (78%)	0.003
Ever got lesions/burns on lips from smoking crack^a^	121 (27%)	45 (30%)	NS
Injection Drug Use	41 (7%)	112 (69%)	<0.001
Mean number of drugs used (±SD)	2.1 ± 1.5	3.3 ± 2.2	<0.001
Drug Treatment Program	334 (54%)	125 (77%)	<0.001
More than 7 drinks over past week	327 (53%)	93 (57%)	NS
Binge drinking	211 (34%)	81 (50%)	<0.001
Attended AA meetings	308 (50%)	112 (69%)	<0.001
**Sexual Behaviors**			
Past history of STD	389 (63%)	103 (64%)	NS
**Sexual History in Past 4 mo**			
Mean number of sex partners (±SD)	3.4 ± 9.8	4.5 ± 12.3	NS
Always used condom	116 (19%)	29 (18%)	NS
Sex under influence of alcohol/drugs	466 (75%)	121 (75%)	NS
Traded sex for drugs, money and other things	274 (44%)	104 (64%)	<0.001
**Potential Risk Factors**			
Number of times shared tweezers/nail clippers (±SD)	21.1 ± 36.0	24.6 ± 37.9	NS
Number of times used toothbrush of other person (±SD)	1.21 ± 7.41	0.8 ± 2.8	NS
Ever gotten a tattoo	154 (25%)	56 (35%)	0.01
Ever gotten a body piercing	569 (92%)	152 (94%)	NS
Ever received a blood transfusion	106 (17%)	54 (33%)	<0.001

HCV: Hepatitis C Virus: STD, Sexually Transmitted Diseases: AA, Alcoholics Anonymous

* Among IDU only, ^a ^Among crack cocaine users only

Women recruited in these studies were sexually active with a mean of 3 - 4 sex partners in the preceding 4 months. Despite reporting several new sex partners in the past 4 months, condom use was low (19%) regardless of HCV Ab status. Both groups reported frequent use of alcohol or drugs before or during their sexual encounters; HCV Ab positive women compared to HCV Ab negative women were more likely to be involved in trading sex for drugs, money and other things. More than 60% of women in both groups reported history of a sexually transmitted disease.

Among other risk factors commonly associated with blood borne infections, both having a tattoo and having had a blood transfusion were found to be significantly associated with HCV Ab positivity in the bivariate analysis. No differences were observed in relationship to body piercings or sharing of personal hygiene equipment.

### Multivariate Analyses

Multivariate logistic regression was performed to identify factors associated with HCV Ab positivity controlling for risk factors associated with HCV transmission in four stages (Table 3**)**. In the first stage, when socio-demographic variables were entered, only older age and history of arrest significantly predicted HCV Ab. Race (African American) and more education were significantly protective of HCV Ab status. In the second stage model, substance use and treatment variables were added to the significant variables from the first stage. In this model, older age remained significant. Additionally, IDU was strongly associated with HCV Ab, with injectors being over 26 times more likely to be HCV Ab positive than non-injectors. The third stage model, which added sexual behaviors, revealed that older women who had history of injection drug use and traded sex were more likely to be HCV Ab positive. In the final stage model, having a tattoo and reporting lifetime crack cocaine use, in addition to age and history of IDU remained significantly associated with HCV Ab positivity. History of blood transfusions displayed a positive trend in association with HCV Ab positivity, but did not reach statistical significance. Overall, IDU remained the strongest predictor.

**Table 3:** Odds ratios for HCV Ab positivity

	***Model 1***		***Model 2***		***Model 3***		**Model 4**	
**Variables**	**OR**	**95% CI**	**OR**	**95% CI**	**OR**	**95% CI**	**OR**	**95% CI**
Never Married	0.80	0.54, 1.20						
Have >1 kids	0.81	0.46, 1.41						
Worked in past 12 months	0.82	0.56, 1.19						
Considered being homeless	1.07	0.70, 1.63						
African American	0.35	**0.21, 0.56**	1.27	0.66, 2.47				
Older (37+ years)	3.81	**2.43, 5.98**	2.96	**1.78, 4.92**	2.78	**1.68, 4.59**	3.04	**1.74, 5.31**
High school education and more	0.60	**0.41, 0.88**	0.78	0.49, 1.26				
Ever been arrested	2.69	**1.53, 4.72**	1.33	0.67, 2.63				
Binge drinking			1.19	0.73, 1.93				
Number of drugs			1.02	0.89, 1.18				
Drug treatment			1.22	0.66, 2.26				
IDU			26.17	**15.19, 45.08**	30.73	**18.70, 50.50**	23.26	**14.31, 37.80**
Attended AA meetings			1.16	0.65, 2.05				
Previous history of STD					0.89	0.54, 1.49		
Sex under influence					0.71	0.40, 1.27		
Number of sex partners in past 4 months					0.99	0.97, 1.01		
Always used condom in past 4 months					1.34	0.73, 2.46		
Trading sex					1.76	**1.06, 2.93**	1.22	0.75, 2.00
Ever gotten a tattoo							2.05	**1.15, 3.66**
Lifetime crack cocaine use							3.87	**1.66, 9.01**
**Ever received blood transfusion**							1.67	0.99, 2.82

OR: Odds ratio, CI: confidence interval, IDU: Injecting drug users, STD: Sexually Transmitted Disease

### Subgroup analyses

In further subgroup analyses, HCV Ab positive women were stratified by injection drug use status: 50 HCV Ab positive women reported no prior IDU and 111 HCV Ab positive women reported lifetime IDU. HCV Ab positive IDU's were more likely to have a tattoo and reported more crack cocaine use (40% vs. 24%, p = 0.05 and 98% vs. 86%, p = 0.002 respectively). When a subgroup of non-injectors was analyzed by their HCV Ab status, the prior history of blood transfusion, lifetime crack cocaine use and trading sex were predictors of HCV Ab positivity status (Table 4).

**Table 4:** Subgroup analyses of risk factors among non-injectors

**Reported Behaviors among Non-injectors**	**HCV Ab neg** ** N = 578**	**HCV Ab pos** ** N = 50**	**P value**
Ever received a blood transfusion	95 (16%)	15 (30%)	0.02
Ever gotten a tattoo	138 (23%)	12 (24%)	NS
Reused crack pipe belonging to others	262 (64%)	31 (72%)	NS
Ever got lesions/burns on lips from smoking crack^a^	104 (25%)	9 (21%)	NS
Lifetime crack cocaine use	411 (71%)	43 (86%)	0.02
Sex under influence of alcohol/drugs	430 (74%)	34 (68%)	NS
Traded sex for drugs, money and other things	249 (43%)	29 (58%)	0.04
Snorted cocaine	102 (18%)	10 (20%)	NS

HCV: Hepatitis C Virus; ^a^Among crack cocaine users only

## Discussion

Compared to the general population, a substantially higher prevalence of HCV Ab positivity in this large community based sample of predominately African American substance abusing females was a key finding. Nearly two thirds of women with HCV antibodies were not aware of their viral hepatitis status; this is particularly concerning given the chronic nature of HCV infection and implies lack of awareness among health care providers and existing barriers to HCV screening in this population.

Past or present injection drug use in these studies was strongly associated with HCV Ab positivity as shown in previous reports [18,34,35]. Although among the HCV Ab positive subjects almost 70% had a history of IDU, 30% reported no IDU despite several questions on injection drug use in three different portions of the structured assessments.

In addition to injection drug use, having a tattoo and history of crack cocaine use were independently associated with HCV Ab positivity after controlling for IDU. Both crack cocaine use and tattoos have been identified as independent risk factors for HCV acquisition in previous reports, especially non-injection drug implement sharing practices [17,20,21,36,37]; however, urban folklore also indicates that a tattoo is a proxy marker for injection drug use and other risk factors. Our study did not assess further details regarding the type or design, or where the tattoos were received; although some reports suggest these factors may play a role in HCV acquisition [38,39].

In a subgroup analysis of this community sample of non-injecting HCV Ab positive females, a number of high risk behaviors (high prevalence of crack cocaine use accompanied by drug implement sharing) as well as high risk sexual behaviors (sex under influence and trading sex) was significantly higher among the HCV Ab positive IDU’s As noted, these risky behaviors have been associated HCV Ab positivity in non-injecting populations [40-42].

There is mounting evidence regarding the increased risk of HCV among non-IDU's suggesting routes of administration and drug implement sharing practices may serve as an alternative HCV transmission pathway[41]. Data regarding the risk of HCV transmission remains conflicting mainly due to limited amounts of research specifically devoted to these HCV associated risks in non-IDU populations and small sample sizes. Since non-injection cocaine related risk was originally reported by C. Conry-Cantilena, *et al*. [18] and attributed to sharing straws while using cocaine intranasally, it has been a subject of discussion [43]. Some investigators were not able to confirm the independent association of intranasal cocaine use and risk of hepatitis C, although their findings highlight other potential iatrogenic sources of the infection [44,45]. Several studies have shown higher rates of HCV Ab positivity in non-injection drug users although subject recruitment may have introduced a selection bias. For example, a study by Koblin *et al*. [19] aimed to recruit non-injection drug users, including non-injection heroin users, who could have been former injection drug users [36]. Rosenblum *et al*. [46] examined a non IDU population based on having a history of less than 5 lifetime injection episodes, although it is evident that even a single injection exposure could theoretically be sufficient for HCV transmission. Studies by Tortu and colleagues [20,21] that validated self reported drug use, with methodology similar to that utilized in the STS/WTW studies, found high rates of HCV among self reported non-injection drug users. Extensive data obtained on drug implement sharing practices suggested that such practices might be a risk factor for HCV transmission [21]. Furthermore, Tortu *et al*. have suggested that intranasal cocaine use could serve as an indirect determinant for other substance use practices leading to HCV transmission, like injection drug use or injection practices that are not typically perceived as IDU (such as skin popping) [20].

Other potential routes of transmission among women in our study, such as tattooing, were significantly associated with HCV Ab status. Non commercial tattooing has been implicated as a significant risk factor in HCV transmission and deserves more detailed prospective investigation [47]. Similarly to other investigators, we did not find an association with hygiene measures, such as sharing toothbrushes or nail clippers, suggested as potential HCV transmission risk factors [17,48].

In our study HCV Ab positivity was not strongly associated with high risk sexual behaviors. Other studies have found that sexual transmission of HCV is associated with high risk sexual practices, including having an IDU sexual partner. To date, the role of sexual behaviors as a risk factor for HCV transmission remains controversial mainly due to the complex determination of the magnitude of the transmission risk which may vary significantly depending on a specific population being studied [49,13,38]. Besides, studies examining HCV transmission seem to frequently involve HIV co-infected women that appear to have a higher HCV Ab prevalence independent of other risk factors [38].

Although our study was not designed specifically for exploring the epidemiology of hepatitis C, it focused on the risk factors associated with HIV that share common routes of transmission with HCV. As a result, these data provided us with very detailed information concerning potential risk factors associated with the transmission of HCV. One of the limitations of this paper is that the data is cross-sectional and, as a result, we are unable to establish the timing of the HCV infection. These two studies were not specifically designed to examine the epidemiology of HCV infection but rather to determine a baseline prevalence of blood-borne infections and to evaluate the HIV intervention techniques.

Another shortcoming of the study design is that a single ELISA testing for HCV infection was utilized. Despite a high HCV risk profile in this population, a small fraction of false positive ELISA results is possible, although this possibility is unlikely to significantly affect the results of our analyses.

Self-reported drug use is another shortcoming of this study which could result in underestimated contribution of IDU to the risk of HCV infection in this population. In order to avoid that, participants were asked about injection drug use several times during the course of the interview and examined for the presence of injection track marks. Majority of studies evaluating epidemiological risk factors have to utilize self-reported measures, the reliability of which have been studied. The two studies primarily targeted hard to reach populations, which are traditionally challenging to recruit and therefore rarely evaluated in a clinical research setting; as a result some respondents were not located or opted to not participate in either of the studies. Nevertheless we feel that the large sample of respondents recruited is representative of the targeted population.

## Conclusions

This study highlights the prevalence of risk factors common for several major blood borne infections in at risk difficult to reach female populations that is commonly understudied and underreported in literature. Our findings strongly support previous epidemiologic studies showing that injection drug use is a major factor contributing to HCV Ab positivity in this large community sample of predominantly African American substance abusing women. Another revealing finding was lack of awareness of HCV Ab status among the study participants. The fact that the majority of women were not aware of their HCV Ab status has significant implications both from the clinical and public health perspective. Programs targeting high risk female populations are needed in order to improve HCV testing and counseling, develop strategies overcoming barriers to care and ensure referrals to comprehensive treatment models that incorporate addressing substance use. Interestingly, our study demonstrated that factors other than IDU were significantly associated with HCV Ab positivity such as having a tattoo and a lifetime history of crack use without injection use. Other potential routes of HCV transmission should be further investigated in large-scale prospective studies particularly among high risk female populations.

## Competing interests

The authors declare that they have no competing interests.

## Authors' contributions

DN participated in the study design, coordination of statistical analysis and drafted the manuscript. ABA participated in the study design, conducted statistical analysis and helped to draft the manuscript. SB performed the statistical analysis and helped to draft the manuscript. CCO participated in organization of the research team, data cleaning process and helped to draft the manuscript. LBC participated in the design and interpretation of the study and provided invaluable guidance on statistical analysis and drafting of the manuscript. All authors had full access to all of the data (including statistical reports and tables) in the study and can take responsibility for the integrity of the data and the accuracy of the data analysis. All authors viewed and approved the final version
